# Application of modified closed biopsy in rabbit model of VX2-transplanted bone tumor

**DOI:** 10.1186/s13018-021-02333-5

**Published:** 2021-03-20

**Authors:** Lei Peng Bai, Jia Xing Lv, Ling Wei Kong, Hai Ying Cao, Yu Jin

**Affiliations:** Department of Orthopaedics, Affiliated Hospital of Chengde Medical College, 36 Nanyingzi Street, Chengde, Hebei 067000 People’s Republic of China

**Keywords:** Bone tumor, Closed biopsy, Tumor infiltration, Biopsy channel metastasis, Elisa, Animal experiment

## Abstract

**Background:**

This study was aimed to explore the application value of modified closed biopsy technique in puncture biopsy of rabbit VX2 transplanted bone tumor model.

**Methods:**

VX2 tumor was transplanted into the bilateral tibia of 30 rabbits through the tibial plateau to make the model of VX2 transplanted bone tumor. Seven days after modeling, the proximal tibia biopsy was performed under the guidance of X-ray, and the biopsy specimen was examined pathologically. The left leg was biopsied with modified closed biopsy technique (experimental group), and the right leg was biopsied with hollow needle (control group). After 14 days of modeling, all rabbits were killed after X-ray examination around the puncture hole, and the soft tissue around the puncture hole was taken for pathological examination, and the expression levels of PCNA and CD34 in the tissue extract were detected by enzyme-linked immunosorbent assay (ELISA).

**Results:**

By the end of the experiment, a total of 3 rabbits died, and finally, 27 rabbits were included in the study. Tumor cells were detected in all the intramedullary specimens obtained by puncture biopsy. On the 14th day after modeling, X-ray showed that the occurrence rate of periosteal reaction and extraosseous high-density shadow around the puncture hole was 14.81% (4/27) in the experimental group and 40.74% (11/27) in the control group. The difference was statistically significant (*P*<0.05). The pathological results of soft tissue around the puncture hole showed that the tumor cell metastasis rate was 29.63% (8/27) in the experimental group and 100% (27/27) in the control group, and the difference was statistically significant (*P*<0.05). The expression levels of PCNA and CD34 in the experimental group were lower than those in the control group (*P* < 0.05).

**Conclusion:**

Both the modified closed biopsy technique and needle aspiration biopsy can provide sufficient biopsy tissue for the diagnosis of VX2-transplanted bone tumor in rabbits. At the same time, the improved closed biopsy technique has a certain application value in preventing local metastasis of tumor cells along the puncture channel.

## Background

Malignant bone tumors account for 6% of malignant tumors in adolescents under 20 years old, of which about 2/3 are osteosarcoma, and most of the others belong to Ewing sarcoma family [1]. Although imaging examination can obtain basic information about the nature, size, anatomical location of the tumor, its impact on surrounding bones or soft tissues, and involvement of adjacent joints and neurovascular structures, the tissue biopsy is the first choice for the diagnosis [2]. Fine needle aspiration biopsy has the advantages of small trauma and high diagnosis rate, which is the main method of closed tissue biopsy at present, but there is the problem of tumor cells spreading along the puncture channel [3, 4]. VX2 tumor originated from human papilloma derived squamous cell carcinoma induced by Shope virus and formed after 72 generations of transplantation [5]. A large number of studies have shown that rabbit VX2 tumor grows rapidly and can be inoculated into the lung, liver, muscle, or bone tissue [6, 7]. Rabbit VX2 bone tumor model has the similar biological characteristics of human malignant bone tumor, which can be used to simulate the proliferation and metastasis process of human bone tumor [8, 9]. PCNA is a good indicator of cell proliferation, and CD34 is a characteristic surface marker of hematopoietic stem/progenitor cells. The combination of PCNA and CD34 can reflect the proliferation of tumor [10, 11]. The purpose of this study was to investigate the application value of improved closed biopsy technique in puncture biopsy of VX2 bone tumor model in rabbits.

## Materials and methods

### Experimental animal

Thirty Japanese big ear white rabbits (hereinafter referred to as rabbits), half male and half female, 3 months old, and weighing 2.0–2.5 kg, were purchased from the Beijing Changyang Xishan farm [animal license No.: scxk (Beijing) 2016-0007]. The rabbits were fed at 25 °C and 50% humidity for 12/12-h light/dark cycle. Access to food and water is limited (2 times per day). This study was approved by animal ethics committee of the Affiliated Hospital of Chengde Medical College (Approval batch number: LL047). The study was conducted in strict accordance with the recommendations of the National Institutes of Health’s guidelines for the Care and use of Experimental Animals.

### Experimental cells and instruments

**Fig. 1:**
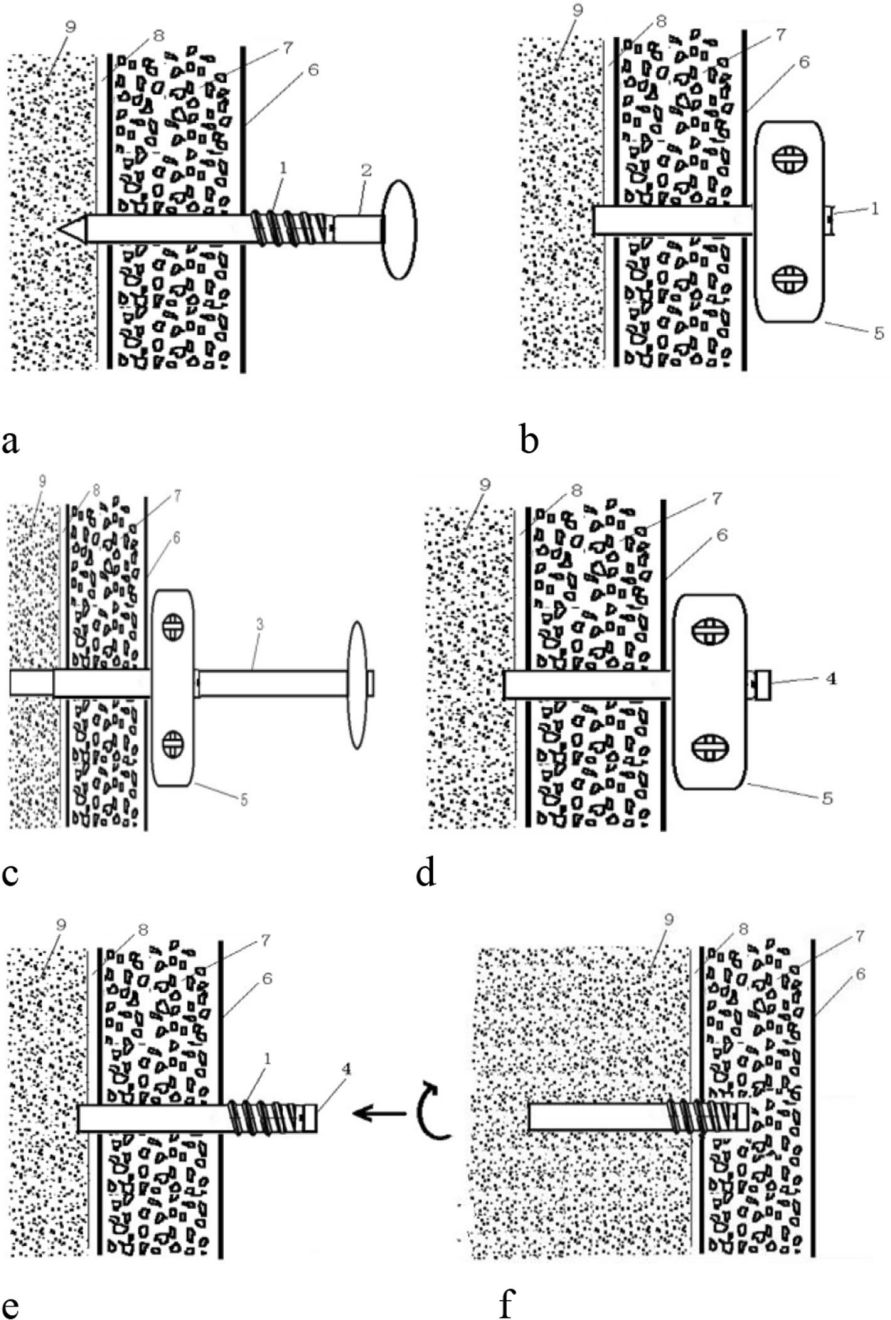
Principle of closed biopsy of bone tumor with built-in channel device. **a** The puncture needle was put into the cannula of the passage and penetrated into the skin, subcutaneous tissue, and bone cortex to enter the tumor site. **b** Fix the puncture sleeve with temporary fixing device. **c** The pathological cannula was used to remove the bone tissue through the passage. **d** After sampling, the tail cap was implanted into the casing to block the puncture hole. **e** Remove the fixator and screw the sleeve under the skin. **f** The built-in channel is fixed to the puncture bone hole, so as to achieve the purpose of blocking the pathological approach. In addition, (1) internal passage, (2) puncture needle, (3) temporary fixator, (4) removal of pathological sleeve, (5) closure of tail cap, (6) skin, (7) subcutaneous tissue, (8) cortical bone, and (9) medullary cavity

**Fig. 2:**
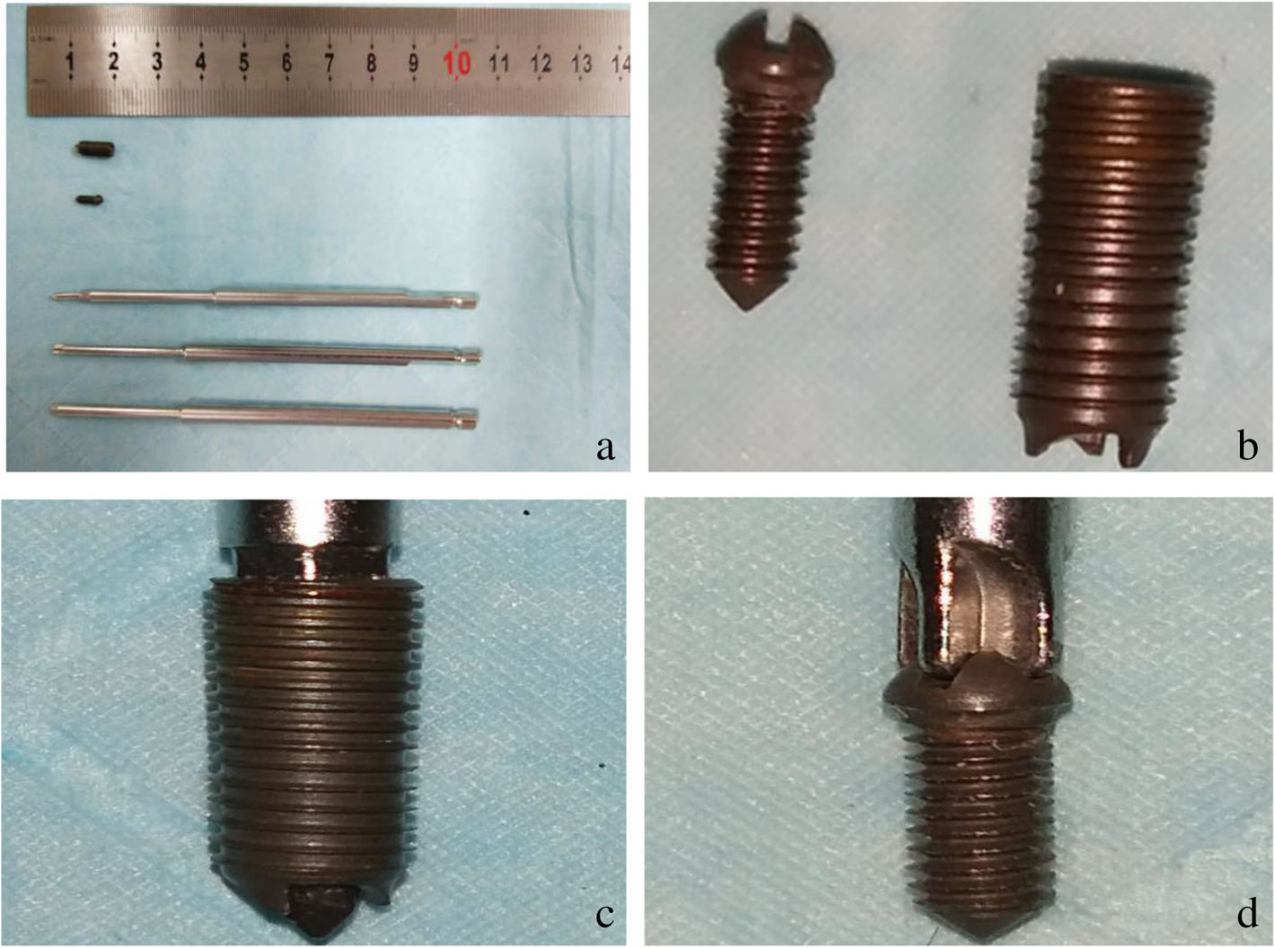
Improved internal channel biopsy device for bone tumor biopsy. **a** The internal channel sleeve, plugging screw, and puncture handle. **b** Built-in channel and plugging tail screw. **c** Built-in channel on puncture needle. **d** Plugging tail screw and handle

The Experimental cells and instruments are as follows: X-ray machine (OEC9800, Shanghai Xianwei Optoelectronic Technology Co., Ltd.); Plate reader (Multiskan FC) which was provided by the Central Laboratory of the Affiliated Hospital of Chengde Medical College; Olympus-BX53 optical microscope (Olympus-BX53, Olympus Company of Japan); bone marrow aspiration biopsy needle [BM09/15, Demeter Medical Technology (Beijing) Co., Ltd.]; VX2 tumor cell line which was purchased from the Chongqing Mengbo Biotechnology Co., Ltd; PCNA, CD34 antibody kit (purchased from Hebei ruipat Biotechnology Co., Ltd.); 1640 cell culture medium and fetal bovine serum(purchased from the Hebei Ruipat Biotechnology Co., Ltd.); and the self-designed and improved channel built-in bone tumor pathological tissue extraction device for closed biopsy (Patent No.: zl201416055578.0) which is made of titanium alloy (Figs. 1 and 2).

### Preparation of VX2 tumor tissue

The cryopreservation tube of VX2 tumor tissue block was taken out from the sealed dry ice box and quickly resuscitated in 37 °C warm water. After being rinsed with PBS for 3 times on the sterile operating table, 1640 cell culture medium containing 10% fetal bovine serum was added for temporary storage. A part of VX2 tumor cells was injected into the left lateral femoral muscle of rabbits for continuous passage culture, and the tumor size was evaluated by color Doppler ultrasound. Four weeks later, when a mass of 3 cm × 3 cm in size was palpable subcutaneously, and anesthesia was performed by intravenous injection of pentobarbital sodium (35 mg/kg). The disappearance of corneal reflex was regarded as the successful sign of anesthesia. The rabbit hair was removed from the tumor area and fixed on the animal experimental table in prone position. The operation area was disinfected with Iodophor, covered with sterile pore towel, skin was cut, and subcutaneous tissue and tumor were separated. After the tumor was completely removed, the tumor was moved to a sterile worktable and washed in PBS at 37 °C for 3 times to remove the blood stain, necrotic tissue, and fibrous tissue in the tumor. The tumor was cut into 1 mm × 1 mm × 1 mm tissue block with ophthalmic scissors. After adding 1640 cell culture medium containing 10% fetal bovine serum, it was stored in refrigerator at − 80 °C. The VX2 tumor tissues used in this experiment were obtained after one passage in rabbits.

### Preparation of rabbit VX2 bone tumor model

Thirty rabbits were anesthetized by intravenous injection of pentobarbital sodium (35 mg/kg). After depilation, the rabbit hind limbs were fixed on the operating table in supine position. The hind limbs were disinfected with Iodophor and covered with sterile hole sheet. A 1-cm incision was made in the inner skin of the knee joint. The subcutaneous tissue and fascia were incised layer by layer to expose the patellar ligament. The patellar ligament was separated longitudinally, and the tibial plateau was exposed. Drill the tibial plateau with Kirschner wire (1 mm in diameter), insert a 2-cm long cannula into the bone hole, push 1 mm^3^ VX2 tumor tissue into the tibial medullary cavity along the cannula, pull out the cannula, and block the bone hole with bone wax. The tissue around the bone foramen was rinsed with absolute ethanol for 3 times and then soaked for more than 1 min each time. After reconstruction of patellar ligament, the incision was sutured layer by layer. After the rabbits were anesthetized, they were put back into the cage to continue feeding. The left leg of all rabbits was set as the experimental group, and the modified closed biopsy technique was used for biopsy; the right leg was set as the control group, and the hollow core needle was used for biopsy. The rabbits in both groups were intramuscularly injected with penicillin 800 000 U/time, once a day, for 3 consecutive days

### Application of closed biopsy technique in puncture

**Fig. 3:**
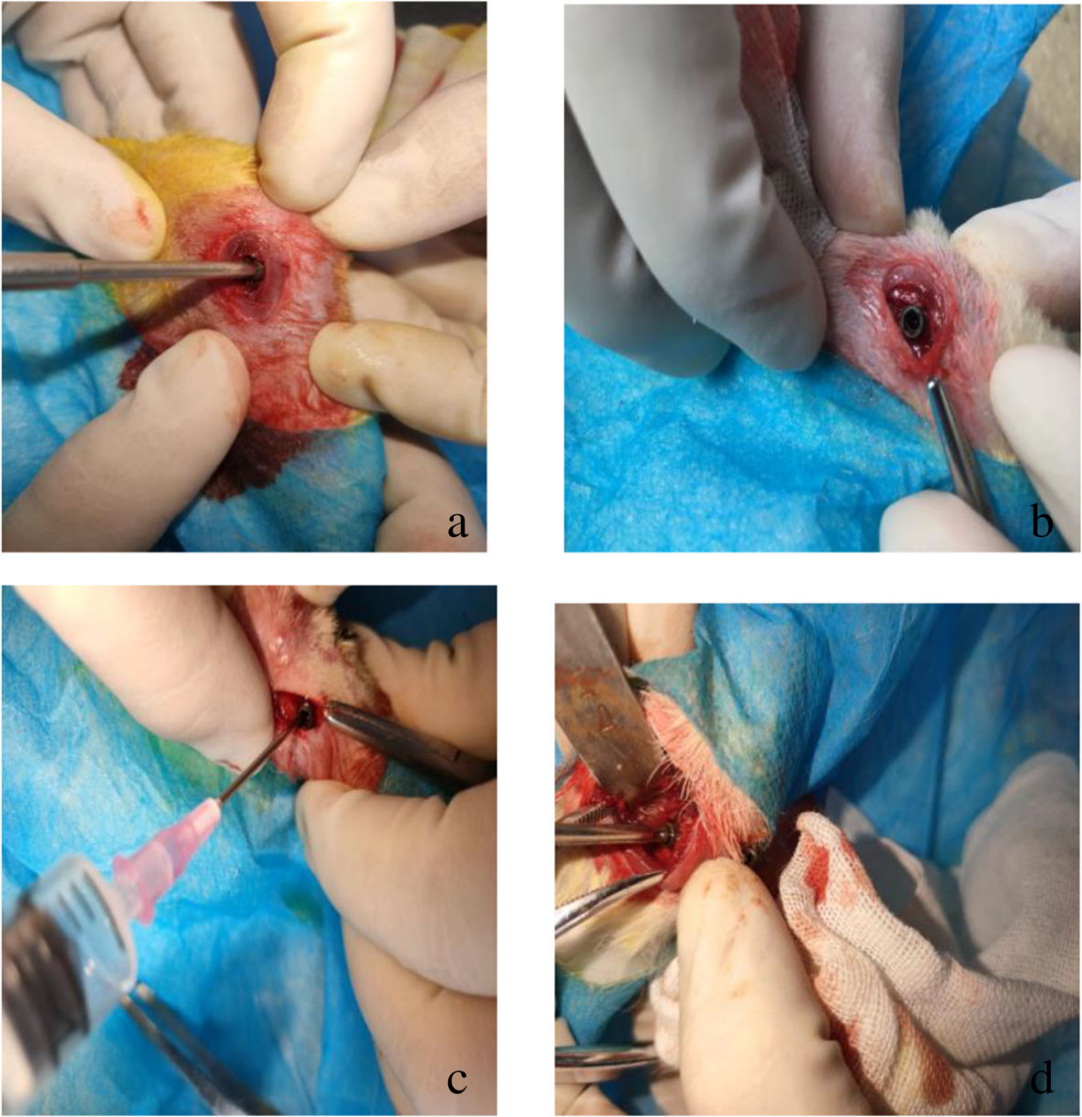
The process of performing a biopsy using a internal channel. **a**, **b** The channel in the puncture handle enters the medullary cavity through the bone cortex. **c** Extracts the contents of the medullary cavity through the built-in channel. **d** The internal channel is blocked by tail nail

Seven days after modeling, rabbits were anesthetized by intravenous injection of pentobarbital sodium (35 mg/kg) through ear edge vein. Under the guidance of X-ray, a 0.5-cm longitudinal incision was made at 1 cm below the left knee joint and the lateral tibia as the puncture point. Install the built-in channel on the puncture handle, penetrate through the bone cortex and enter into the medullary cavity. Pull out the puncture handle. The built-in channel remains in the bone cortex to form an artificial channel. After extracting 2 ml of pulp cavity contents through the built-in channel with G14 syringe needle, the tail nail is screwed into the internal channel (Fig. 3) to achieve the effect of closing the puncture channel and suture the incision layer by layer. The same method was used in the diagnosis of bone tumor with hollow core needle aspiration biopsy in the right tibia. One centimeter below the right knee joint and the outside of the tibia was used as the puncture point. After exposing the bone surface, the bone marrow puncture needle was used for puncture biopsy. After obtaining a 2-ml bone marrow sample, the puncture hole was not sealed, and the puncture hole was compressed to stop bleeding, and the incision was sutured layer by layer. After the puncture of bone tumor was completed, the rabbits were put back into the cage to continue feeding. The rabbits in both groups were intramuscularly injected with penicillin 800 000 U/time, once a day, for 3 consecutive days.

### Imaging and pathological examination

Fourteen days after modeling, X-ray examination of bilateral tibia, intraperitoneal injection anesthesia (pentobarbital sodium, 40 mg/kg), and ear vein air embolism were performed to kill the rabbits, with the disappearance of palpable chest heartbeat as the death mark. The soft tissue within 2 cm around the puncture hole was resected. Hematoxylin eosin staining (HE staining) and microscopic examination were performed. The results of microscopic examination were performed by the same senior pathologist with blind method.

### Elisa examination

A 0.1-mg tissue slice was transferred into a glass grinder on the sterile operating table. PBS (1 mg: 9 ml) was added to grind it fully, centrifuged at 4000R/min for 5 min. The supernatant was collected. The content of PCNA and CD34 in the tissue supernatant was determined by ELISA. The test process was carried out in strict accordance with the instructions of the kit.

### Statistic analysis

SPSS 25.0 (SPSS Inc., Chicago, IL, USA) was used to analyze the data. Chi-squared test was used to analyze the results of imaging and pathological examination 14 days after modeling. The expression levels of PCNA and CD34 in the soft tissue around the puncture hole of VX2 bone tumor were analyzed by paired samples *t* test. *P* < 0.05 was statistically significant.

## Results

### Modeling and biopsy results

In 30 rabbits, one died of shock 1 day after bone marrow puncture, and two died of multiple organ failure 9 days after modeling. A total of 27 rabbits were finally included in the study. There was no obvious infection in the incision of model implantation and biopsy, but the growth and healing of skin tissue was slow. Tumor cells were detected in the biopsy tissues of 27 rabbits, and the tumor diagnosis rate was 100.00%.

### Imaging and pathological examination results

**Fig. 4:**
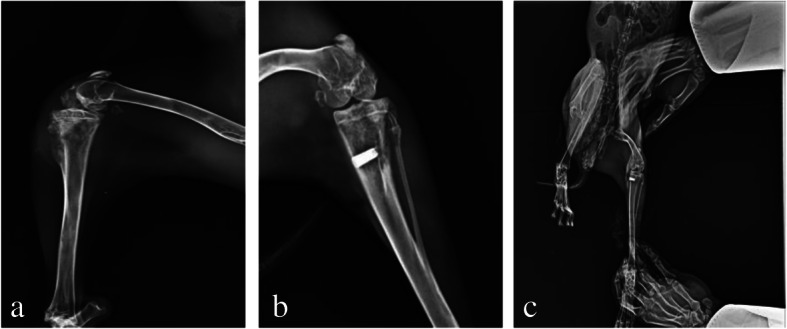
X-ray of tibia around puncture passage on the 14th day of modeling. **a** In the control group, periosteal reaction and a large number of irregular high-density shadows could be seen around the puncture hole. **b** In the experimental group, no obvious abnormal image was found in the soft tissue around the puncture hole, and periosteal reaction and calcification were seen in the contralateral cortex

**Fig. 5:**
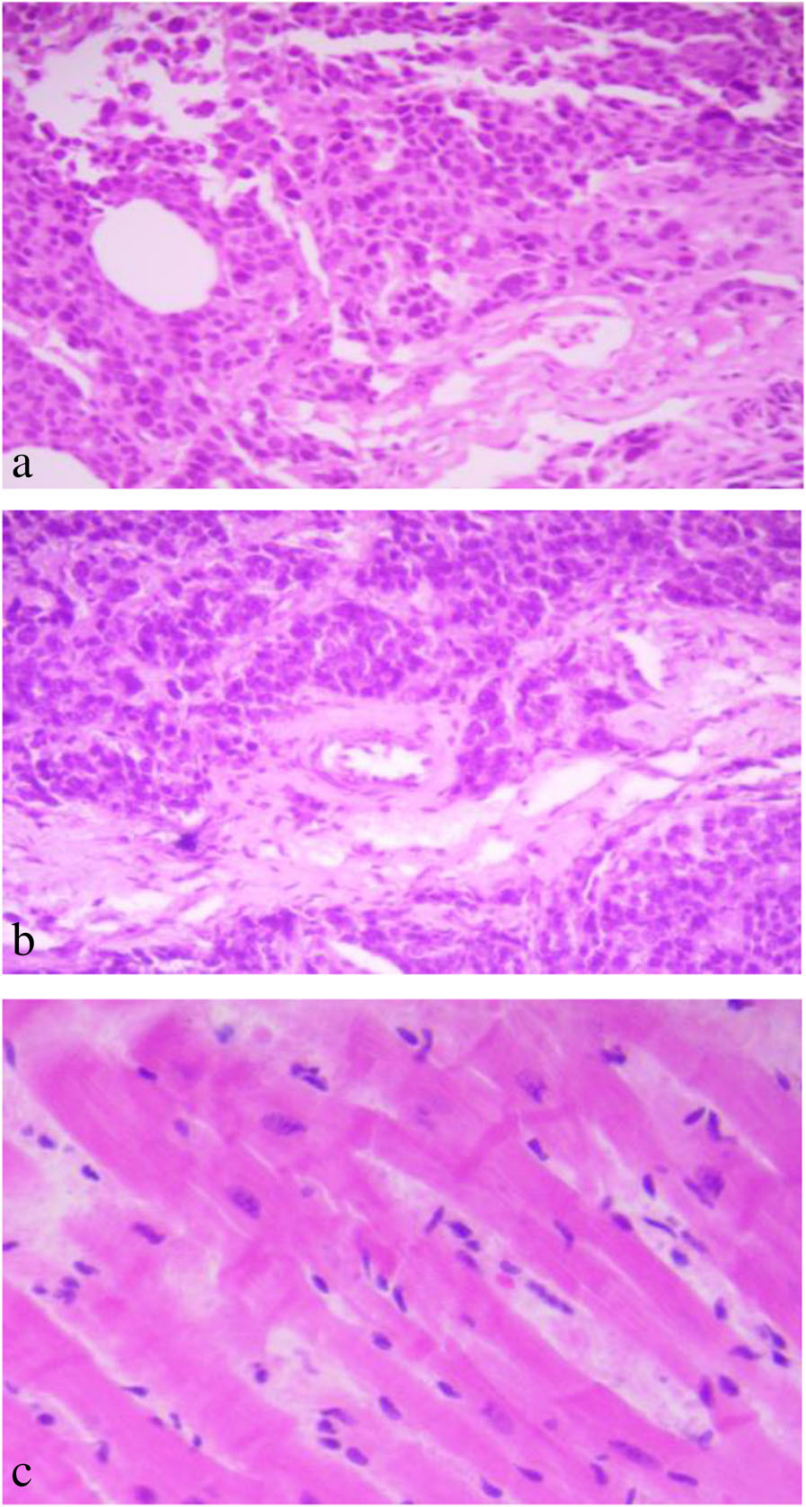
The tissue obtained by biopsy and the pathology of soft tissue around the puncture hole on the 14th day of modeling (HE × 40). **a** The intramedullary tissue was obtained by puncture. Microscopically, a large number of fat vesicles and tumor cells were found, with large volume, irregular shape, enlarged nucleolus, and deep staining. **b** In the control group, a large number of irregular muscle tissue and tumor cells infiltrated into the soft tissue around the puncture hole. **c** Normal muscle tissue outside the puncture hole in the experimental group

Fourteen days after modeling, X-ray examination results showed that periosteal reaction and high-density shadow around the tibial puncture hole appeared in 4 rabbits in the experimental group and 11 rabbits in the control group (Fig. 4). All the built-in channels were in good position at the puncture hole. The incidence of periosteal reaction and high-density shadow around the puncture hole in the experimental group was 14.81% (4/27) and that in the control group was 40.74% (11/27), the difference was statistically significant (χ^2^ = 35.092, *P* = 4.514e−9 < 0.05). The pathological results of the soft tissue around the puncture hole showed that tumor cells were detected under microscope in 8 rabbits in the experimental group and 27 rabbits in the control group (Fig. 5). The metastasis rate of tumor cells in the experimental group was 29.63% (8/27) and that in the control group was 100.00% (27/27), the difference was statistically significant (χ^2^ = 29.314, *P* < 0.05).

### ELISA results

The expression level of PCNA in the soft tissue around the puncture hole in the experimental group was 73.85 ± 13.13 ng/ml, and that in the control group was 81.93 ± 15.16 ng/ml, the difference was statistically significant (*t* = −2.52, *P* < 0.05). The expression level of CD34 in the soft tissue around the puncture hole in the experimental group was 75.68 ± 9.86 ng/ml, and that in the control group was 81.03 ± 6.67 ng/ml, the difference was statistically significant (*t* = −2.72, *P* < 0.05).

## Discussion

In recent years, the treatment of malignant tumors has become a worldwide medical problem. The incidence rate of bone tumors is increasing year by year [1]. Appropriate and timely diagnosis and treatment methods of multidisciplinary cooperation are very important to improve the treatment results of tumor. The final diagnosis results of tumor need to be combined with the detection results of clinical manifestations, imaging, pathology, and molecular biology [10, 11]. Accurate pathological diagnosis is helpful to choose appropriate treatment plan and prognosis. At present, tissue biopsy is still the “gold standard” for clinical diagnosis of tumor [12], which requires the correct design and implementation of experienced team. The bleeding of puncture channel and tumor cell proliferation after biopsy have gradually attracted the attention of clinicians, and it has gradually become a consensus to resect or block the puncture channel [13].

The methods of tissue biopsy include closed biopsy, open biopsy, and excision biopsy. Among them, the advantages of incision biopsy are to obtain sufficient tumor samples and high diagnostic accuracy [14], while the disadvantages are bleeding and local tumor cell proliferation. In addition, the incision may affect the subsequent surgical treatment. Closed biopsy includes fine-needle aspiration biopsy and hollow core needle biopsy, with the advantages of small trauma, less bleeding, and simple operation, which is the most commonly used method by orthopedics doctors; the disadvantage is that the tumor samples obtained are small [15]. After biopsy, the puncture hole needs compression hemostasis or bone wax plugging, and the puncture hole will naturally close under the natural healing of the body tissue [16]. But before that, tumor cells may spread to the outside of the puncture hole under the effect of pressure difference between inside and outside the medullary cavity. Existing tissue biopsy techniques can damage the natural barrier around the tumor, resulting in exudation or tumor cell proliferation [17–20]. Barrientos et al. [13] found that the implantation rate of tumor in the puncture channel was 0.8%.

Proliferating cell nuclear antigen (PCNA) is a kind of circular homotrimer, which is mainly involved in the coordination of DNA replication and repair. PCNA surrounds and slides along the DNA in the nucleus. DNA polymerase, helicase, exonuclease, ligase, cell cycle regulator, acetyltransferase, chromatin reconstitution, and histone chaperone interact with DNA by binding with PCNA [21]. PCNA was expressed in all cell cycles of normal tissues, and its expression level was relatively stable [22]. Some rabbit experiments [23] showed that the level of PCNA expression was significantly reduced by the stimulation of traumatic factors. CD34 is a highly glycosylated type I transmembrane protein, which is selectively expressed on the membrane of hematopoietic stem cells and/or progenitor cells. It is a stage-specific rather than a series specific cell membrane surface antigen. It is more expressed on immature hematopoietic stem cells. The expression rate of CD34 antigen in the earliest hematopoietic stem/progenitor cells is the highest, which gradually decreases with the differentiation and maturation of cells, until it disappeared [24, 25]. Therefore, HE staining and the expression of PCNA and CD34 were used to evaluate the infiltration degree and proliferation rate of tumor cells in the peripheral soft tissue of puncture passage.

In this study, the built-in channel biopsy instrument used to reduce the proliferation of tumor cells along the puncture channel is a patented product of our research group (China Patent No. zl201416055578.0). The built-in channel and plugging screw are made of titanium alloy material which can be implanted into the human body and can be retained in the human body for a long time. Enough pathological tissue was obtained by inserting metal channel and suction, and the puncture channel was closed by screw in closure screw, so as to reduce local exudation and tumor cell proliferation. The rabbit VX2 bone tumor model used in this study is a commonly used animal model for bone tumor research [26–28, 29, 30]. Previous studies have shown that in the first week after VX2 tumor cell transplantation, the tibial tumor grew slowly in the medullary cavity with intact bone cortex; at the second week, the tumor could basically grow to the edge of the bone cortex; at the third week, X-ray film showed that the tumor grew rapidly and the bone cortex was partially destroyed, but it would not appear in the surrounding soft tissue [31]. In order to eliminate the influence of tumor on the surrounding soft tissue and make the experimental process more in line with the clinical diagnosis process, a 1 week model was used in this study. The difference between the two groups was statistically significant, indicating that the improved biopsy method can effectively prevent tumor proliferation.

To sum up, the improved closed biopsy technique not only physically isolated the bone tissue and peripheral soft tissue of the puncture channel, but also blocked the puncture hole. The whole device is made of titanium alloy which can be implanted into organism and can be stably fixed in the puncture hole to prevent the tumor from spreading. The results of this study indicate that the use of the built-in channel biopsy technique in rabbit VX2 bone tumor model can reduce the tumor proliferation of the tibial puncture hole.

This study has some limitations: compared with the rabbit body size, the instruments used in the experiment are larger, so the two puncture methods can detect 100% tumor cells. However, the puncture channel left by core biopsy was also large, which led to the high infiltration rate of soft tissue tumor around the puncture hole in the control group, and the data was biased due to the small sample size. However, due to the limitation of metal puncture channel, the direction of taking materials is limited, and false negative results may appear in the application of the closed biopsy instrument. Moreover, the applicability of the device to the indwelling site, the influence on bone strength, and whether long-term indwelling in the body will lead to rejection reaction need to be further studied. The research group will continue to improve the design of the device and carry out further research to provide more reference for clinical diagnosis.

## Conclusions

In summary, our study reveals that both the modified closed biopsy technique and needle aspiration biopsy can provide sufficient biopsy tissue for the diagnosis of VX2 transplanted bone tumor in rabbits. At the same time, the improved closed biopsy technique has a certain application value in preventing local metastasis of tumor cells along the puncture channel.

## Data Availability

The datasets used and/or analyzed during the current study available from the corresponding author on reasonable request.

## Abbreviations

PBS: Phosphate-buffered saline
HE: Hematoxylin-eosin staining
ELISA: Enzyme-linked immunosorbent assay
PCNA: Proliferating cell nuclear antigen
CD34: Hematopoietic progenitor cell antigen 34

## Declarations

The authors confirm that all the experiments described in this study have been carried out and disagreed with any criticisms or requested revisions.
